# Endocannabinoid system components as potential neuroimmune therapeutic targets in tinnitus

**DOI:** 10.3389/fneur.2023.1148327

**Published:** 2023-05-25

**Authors:** Vishweshwara Bhat, Emmanuel Shan Onaivi, Venkatanarayanan Sharma

**Affiliations:** ^1^Speech Language Pathology, William Paterson University, Wayne, NJ, United States; ^2^Biology Department, William Paterson University, Wayne, NJ, United States

**Keywords:** cannabinoids, endocannabinoids, cytokines, COVID-19, neuroinflammation, tinnitus

## Abstract

Research interest in understanding tinnitus has increased severalfold in the last decade to find a cure for this auditory disorder. Hyperacusis can also accompany tinnitus, although the mechanisms involved in hyperacusis and tinnitus are different. Millions of people suffer from some degree of tinnitus with hearing loss. Tinnitus is believed to be a form of sensory epilepsy, spawning neuronal hyperactivity from the cochlear nucleus and inferior colliculus of the auditory brainstem region. Cannabis has been used for recreation, medicinal purposes, and served as an entheogen from time immemorial. With the current and increasing global medical and recreational cannabis legalization, there is renewed enthusiasm for the use of cannabinoid drugs, and the role of the endocannabinoid system (ECS) in several health disorders including tinnitus which is associated with COVID-19. The ECS signaling pathways have been proposed to affect the underlying pathophysiology of tinnitus. Cannabinoid receptors (CBRs) have been found in the auditory system, raising interest in ECS signaling in hearing and tinnitus. However, previous studies mostly in animal models of tinnitus did not investigate the involvement of CB2Rs but focused on CB1R-based responses, which suggested that CB1R ligands had no effect and may even be harmful and worsen tinnitus. With new molecular techniques and transgenic approaches used to dissect the complexity of the ECS, the role of ECS/CB2R neuroimmunological function in the auditory system and tinnitus is emerging. This perspective proposes the role of emerging neuroimmune crosstalk of the ECS in sound-sensing structures of the auditory system as a potential pharmacogenomic therapeutic target using cannabinoid CB2R ligands in tinnitus in the era of the COVID-19 pandemic.

## Introduction

Tinnitus can be induced by disruption of the complex network of neuroimmune peripheral and central auditory pathways and may be accompanied by hyperacusis—a hypersensitivity to noise, although the mechanisms involved in hyperacusis and tinnitus are different. Tinnitus can disrupt the quality of life of some patients and is the perception of “source-less” noise in the ear(s) when no external sound is present. There are two types of tinnitus, and most people with tinnitus perceive a wide variety and range of sounds that could be static or high-pitch tones such as ringing, hissing, buzzing, grinding, whooshing, screeching, sirens, clicking dial tones, and even music. Such subjective tinnitus characterized by “phantom” sounds is most common due to inner ear damage and loss of sensory hair cells and receptors in the cochlea, disrupting neuronal plasticity and auditory transduction (1). Unlike the subjective form of tinnitus in which only the individual can hear the sound, in objective tinnitus (somatosounds), someone nearby can hear the sound, which can be detected by stereoscopic clinical examination. Objective tinnitus is a rare type that is less common and is predominantly due to peripheral vascular abnormalities, such as physiological disorders that affect nerves, muscles, or blood vessels. Furthermore, spontaneous otoacoustic emissions (SOAE) have been described as another form of “objective tonal tinnitus”, as distortion-product otoacoustic emission amplitudes are reduced in patients with tinnitus (2).

Sensorineural hearing loss (SNHL) can co-occur with tinnitus in both or the affected ear due to reduced sound transmission to hearing nerves in the brain. However, genomewide association studies (GWAS) and transcriptome-wide analysis studies (TWAS) in mice and humans do not correlate but have been reported that both hearing loss and tinnitus are polygenic disorders (3). In any case, tinnitus can be characterized by its intensity and frequency content that is gradual or instant onset, could be mild to severe, temporary or persistent, and permanent (4).

### Pathophysiology of tinnitus

The pathophysiology of tinnitus remains challenging, unclear, and poorly understood, and its diagnosis and treatment are complicated by its polygenic nature, with different associated mechanisms in the auditory pathways (3). This is because increased somatization and functional changes in auditory and non-auditory areas are involved in the pathophysiology of tinnitus (5, 6). The global variable prevalence of tinnitus is 5–43% (6), which is influenced by many factors, including age, gender and race/ethnicity, exposure to intense sound, or acoustic trauma. This prevalence has been associated with socioeconomic, lifestyle habits (loud music noise), and anthropometric characteristics in human populations (7). Such a global wide range of prevalence data has been associated with a lack of a simple definition of tinnitus (6). Noise-induced hearing loss, which may be accompanied by tinnitus and hyperacusis, causes inflammation because of the immune system's response to injury or infection in the central auditory pathway. Tinnitus can be induced under stressful or emotionally depressing situations where there is no presence of an auditory insult. In these conditions, tinnitus may disappear when the emotional factors become normal. However, tinnitus with noise-induced hearing loss seems to be permanent in most cases.

In addition, the worldwide prevalence of tinnitus is reported to have increased by the COVID-19 pandemic (8). In some cases, it appears that tinnitus associated with COVID-19 infection does not differ from the pathophysiology of tinnitus before the COVID-19 infection. However, there are inconsistent results, as reported in older adults, that the hearing difficulties may be a misperception due to wearing masks (9). Not surprisingly, the quality of life may be impacted significantly by tinnitus, which can induce irritability, anxiety, pain, depression, and mood swings. Tinnitus can also co-occur with diseases like otosclerosis, where the stapes have reduced mobility due to the calcification of the footplate in the middle ear (10). In addition to aging, noise, trauma, stress, depression, and several medications can also cause tinnitus (11). In Meniere's disease, a condition where there is an overproduction of endolymph causing pressure in the cochlea, tinnitus has been observed. Ototoxic drugs, including salicylates, other NSAIDs, and benzodiazepines can trigger the most common type of high-pitch tinnitus, especially when administered at high dosages for a prolonged time. Other than noise-induced tinnitus, several clinical studies report that pressure on the acoustic nerve can result in tinnitus most commonly observed in one ear. There is evidence of a physiological mechanism for tinnitus in unilateral sporadic vestibular schwannoma, characterized by tiny growths on the vestibule-cochlear nerve (12). These tiny growths on the myelin sheath on the acoustic nerve induce pressure, thereby causing tinnitus. The audiogram in those patients shows sensory loss due to cochlear involvement (12).

While the causes of tinnitus are not completely understood, some of the common causes are known, with an established dogma, that tinnitus can be characterized as a syndromic symptom, indicating some underlying causes from various pathologies. Tinnitus may accompany many different health disorders such as epilepsy, neuropathic pain, anxiety, and stress that are known to be responsive to cannabinoids (13). However, accumulating evidence from animal and clinical studies suggests that auditory brain regions are involved in the maintenance of tinnitus-generating neural activity triggered by damage to the cochlea (1, 4) and cochlear synaptopathy. Therefore, the emerging neuroimmunological basis of tinnitus from animal and epidemiological studies, and psychoacoustic measures in humans, support a new paradigm-shifting concept of tinnitus heritability with single nucleotide variants that have been reported in tinnitus patients but not in individuals without tinnitus (14, 15). This new evidence that tinnitus is a complex polygenic disorder coupled with the growing knowledge of the neuroimmunological basis of alteration in the auditory neuraxis may unravel new pharmacogenomic therapeutic targets in silencing tinnitus. The relationship between cannabis use and tinnitus is complex with the ECS expressed in the auditory system playing a regulatory role in the cochlear nucleus. Regular cannabis use is associated with tinnitus in some patients (16), although no dose–response between cannabis use and tinnitus has been established. However, in their studies Ghosh et al. (17) demonstrated a protective role of the ECS/CB2R system, requiring a CB2R agonist boost in maintaining normal hearing in the rat model of cisplatin-induced hearing loss.

### The impact of the COVID-19 pandemic on tinnitus

The global pandemic of COVID-19 caused by the severe acute respiratory syndrome coronavirus 2 (SARS-CoV-2) remains a widespread infection with initial fever, fatigue, cough, dyspnea, anosmia, and ageusia symptoms and some with cerebrovascular-neurological dysfunction (18–20). Several studies and reviews have now documented the initial gateway through respiratory cells and spread to other organ systems leading to many disorders including audiovestibular, neuropsychiatric, and neurological manifestations (8, 9, 18, 20–30). COVID-19 and the evolving variants of the SARS-CoV-2 virus, some with viral escape mechanisms for immune evasion from vaccines and immunity from previous infection (31), with long-term sequelae and long haulers symptoms, are associated with the audio-vestibular manifestation of COVID-19 post-viral syndromes (32, 33). Evidence is provided by the detection of the SARS-CoV-2 virus isolated from the mastoid and middle ear and the detection of otolaryngological symptoms, including tinnitus in COVID-19 patients and COVID-19 decedents (27–29, 34). Hospitalized and non-hospitalized COVID-19 patients continue to experience poor life quality some with neurologic and emotional symptoms and tinnitus, although infrequent after vaccination (18, 28–30, 35). However, new-onset tinnitus has been reported in the absence of hearing changes after infection with COVID-19 (26). It should be pointed out that some studies indicate that anxiety and stress from the pandemic contribute to tinnitus (22). Nevertheless, auditory-related symptoms accompany viral infections, such as mumps, measles, meningitis and Guillain–Barre syndrome, tinnitus, and other diverse audio-vestibular symptoms, have been reported in some COVID-19 patients, and coronaviruses can damage the nerves that carry information to and from the brain (27, 28).

Evaluation of post-COVID-19 anamneses is beginning to reveal wide-ranging relapsing–remitting long-haulers syndrome including otolaryngological symptoms and other neurologic manifestations such as “brain fog”, memory, and attention problems. The multifactorial etiologies of SARS-CoV-2 long-haulers symptoms and dysautonomia may be due to “a neuro-immune inflammatory cytokine storm” with no current treatment for long-term post-COVID-19 symptoms that may be more common in women. Long-hauler COVID-19 patients continue to experience lingering symptoms including tinnitus, dizziness or vertigo (24, 25), and vestibular cochlear manifestation in COVID-19 cases (23) even after the virus is gone. Thus, the viral immune evasion from vaccines and antibody resistance can impede COVID-19 vaccines due to the evolution of resistant SARS-CoV-2 variants (36). Understanding the immunological memory of SARS-CoV-2 infection that induces inflammation due to immune response and “cytokine storm” (19) provides a basis for investigating the use of cannabis in quelling the cytokine storm and whether ECS components could be potential therapeutic targets in tinnitus. This is supported by cannabinoid-induced immunomodulation that has been linked to viral infections. Together with the identification of inflammation and that neuroinflammation mediates noise-induced tinnitus in rodent models, the presence of ECS in the auditory system provides a basis for further studies on the evaluation of ECS/CB2Rs neuroimmune signaling in tinnitus, especially as COVID-19 exacerbates tinnitus in some patients.

While respiratory and cardiovascular disorders have been identified as the first initial disease manifestation of COVID-19, tinnitus and other audiovestibular symptoms occur and affect life activities post-COVID. Research has shown that the ECS is widely distributed and involved in peripheral and central auditory pathways in mice and human subjects. Furthermore, several studies and trials indicate that cannabinoids play a useful role in suppressing inflammation in many diseases. Therefore, our rapidly expanding knowledge that neuroinflammation is emerging as a key component in the effects of CB2Rs warrants investigation of the neuroimmune activity of ECS/CB2Rs and the possible use of CB2R ligands in tinnitus with or without COVID-19 infection. Although the pathophysiological model for the origin of tinnitus from animal studies and possible treatment plans are questioned, more studies demonstrate the applicability of models and link these positive results to patient care (37–39). However, it appears that the continuation of more controlled dose-specific studies is needed to confirm the usefulness of cannabinoids as a treatment for tinnitus. It is believed that profound reorganization happens in the brain as a response to any noise assault. When there is an intense sound exposure resulting in damage to the hair cells in the basal part of the cochlea, the auditory pathway, the brain produces abnormal nerve signals to compensate for the missing output perhaps by activation of inflammatory cytokines (40). There is increasing evidence for the CB2R being present in inner hair cells (IHC), outer hair cells (OHC), pillars structures of the organ of corti (OC), stria vascularis (SV), and spiral ganglia (SG) of the basilar membrane in the cochlear nerve (17). In general, neural activities are controlled by CB1R, which is abundant in the brain. However, CB2R is predominantly visible in the immune cells and modulated by inflammatory cytokines. In the case of noise trauma, cytokines are believed to be triggering inflammation, causing auditory neural hyperactivity—a leading cause of tinnitus (40).

The maladaptive lesion-induced hyperactivity in the central auditory pathway provides an opportunity for us to explore using our conditional knockout (cKO) animal models. We believe that significant progress has been made in understanding the neuroimmunological mechanisms of cannabinoid therapy and provides a basis to explore its efficacy in unexplored CB2R cKO mouse models of tinnitus. Such studies will provide us clues to the role of TH1 and TH2 cytokines in the induction of tinnitus. However, controlled clinical trials are needed to prove the efficacy of blocking neuroinflammatory cytokines as possible drug targets for tinnitus. As we learn more about the ECS and with the increasing global use of cannabis, our rapidly expanding knowledge may uncover the neuroimmunological basis of the protective and pharmacotherapeutic potential of CB2R cannabinoid ligands in the peripheral and central auditory pathways associated with tinnitus that require more studies and trials.

Gosh et al. (17) concluded by studying a rat model that the activation of CB2R in the cochlea reduced the damage of sensory cells (IHC and OHC) caused by noise trauma. We hypothesize that the lesser the damage to sensory cells, the greater the possibility of reducing the phantom perception of tinnitus. In addition, we propose that the protective role of CB2R will control tinnitus perception by blocking inflammatory cytokines. Therefore, we conceptualize that by activation of either CB2R directly, or by identifying and suppressing inflammatory cytokines in the auditory nerve, fibers can become attractive targets for tinnitus treatment. Furthermore, the available studies on the pathophysiology of tinnitus and other audiovestibular disorders caused by COVID-19 are not well understood; therefore, more studies are required to investigate the role of CBD (41) and the ECS components as potential neuroimmune therapeutic targets in tinnitus and other vestibular disorders.

### Endocannabinoid system components as potential therapeutic targets in tinnitus

With the current and increasing global medical and recreational cannabis legalization, there is renewed enthusiasm for the use of cannabinoid drugs in several health disorders including tinnitus (1, 13, 42). The ubiquitous ECS consists of enzymes for the synthesis and degradation of endocannabinoids that activate cannabinoid receptors (CB1Rs and CB2Rs) and other candidate receptors (19). The ECS is involved in maintaining neural and/or immune homeostasis in all cells and tissues in mammalian biology and is widely distributed in auditory neuraxis. There is increasing attention on the use of cannabinoids in otolaryngologic pathologies as they may alleviate nausea, vomiting, and pain, but their use in vertigo and tinnitus is controversial (13, 42). However, the functional role and mechanism of ECS involvement in the auditory system tonotopic organization from the cochlea to the central auditory centers have not been completely characterized and mapped. Nevertheless, some studies have found components of the ECS in the primary transducing key sound-sensing structures in the inner ear and central auditory pathways that have been traditionally associated with the neural basis of the genesis of tinnitus. ECS signaling pathways have been proposed to affect the underlying pathophysiology of tinnitus (43). To understand the therapeutic potential of cannabinoids in tinnitus, it is essential to study the relationship between cannabis and the role of components of ECS in signaling pathways associated with tinnitus. This is because the ECS is crucial in brain development and involved in the formation of synapses, synaptic plasticity in the auditory system, and regulation of neurotransmission by retrograde signaling. In retrograde signaling, the excitation of a postsynaptic neuron triggers the synthesis of anandamide and/or 2-AG that travels back to the presynaptic membrane and binds to CB receptors to reduce neurotransmitter activities. Neural hyperactivity has long been associated with tinnitus and the distribution of components of ECS in the auditory system where molecules of exogenous cannabinoids can interact to control neural hyperactivity has been mapped in the cochlear nucleus and inferior colliculus (40).

Interestingly, many previous studies mostly in animal models of tinnitus did not investigate the involvement of CB2Rs but focused on CB1R-based responses, which suggested that CB1R ligands had no effect and may even be harmful and worsen tinnitus (1, 13). With new molecular techniques and transgenic approaches used to dissect the complexity of the ECS, the role of ECS/CB2R neuroimmunological function in the auditory system and tinnitus is being explored. The diverse and complex interactions of the ECS with physiological systems depend on their cellular and tissue distribution patterns throughout the body with similar, differential and sometimes opposing roles of the CB1Rs and CB2Rs. There are limited therapeutic approaches for subjective tinnitus with no efficacious drug treatment, or with some treatments reducing the persistence for some, but none is effective for everyone. Since there is no known cure for tinnitus, there is intensive research to search for novel therapies and targets. Hence, there is renewed interest in investigating the use of cannabis formulations or their components in many disorders including tinnitus. This is because of the anecdotal evidence that tinnitus patients are self-medicating with the easy access and legalization of cannabis and cannabinoid products for relief from the “phantom sounds”. As reviewed by Perin et al. (13), the association between tinnitus and marijuana use in humans has been studied with mixed results. Investigators have extensively studied the origin of tinnitus by focusing on the areas of peripheral and central auditory pathways and some non-auditory regions of the brain (37, 44). While there are no controlled human studies on the effects of cannabis on tinnitus, the implications related to reducing tinnitus-using cannabinoids have been evaluated in animal models of tinnitus (1). Among phytocannabinoids, the best researched are delta-9-tetrahydrocannabinol (Δ^9^-THC), which is the major active component in marijuana responsible for the psychoactive effect, and cannabidiol (CBD), a non-intoxicating phytocannabinoid that is gaining intense focus and research for use in several disorders. Successful use of CBD with approval by the US FDA for the treatment of some rare forms of pediatric epilepsies in children 2 years and older and several trials and studies are underway for other psychiatric disorders. However, the use of cannabinoids to alleviate tinnitus stemming from long-term intense noise exposure is less documented and investigated. CBD appears to be a multi-target drug, and the molecular mechanisms of action of CBD in many psychiatric disorders are of major research interest. The effect of exogenous cannabinoids with a ratio of 1:1 of CBD and THC on tinnitus induced in rats with noise exposure was examined (1, 40). The results agree with the hypothesis that cannabinoid receptors can reduce neural hyperactivity. The role of ECS in hearing abilities was measured in CB1R-knockout mice and compared to those of wild-type mice, and the results suggest that CB1R is involved in auditory processing and lays the groundwork for future physiological experiments (45). However, there is not much information on the role of CB2R and tinnitus and we will be attempting to delineate the molecular mechanism using cannabinoid receptor floxed mouse lines that we have generated using Cre-LoxP recombinant technology (46) to evaluate the functional role of ECS/CB2R neuroimmune axis in a tinnitus model.

### Neuroinflammation, cytokines, and neural hyperactivities in tinnitus: role of ECS/CB2Rs

Several studies indicate that cannabinoids play a useful role in suppressing inflammation in many diseases. Accumulating evidence suggests that the ECS is involved in the regulation of cell proliferation, differentiation, and survival with different outcomes from pluripotent stem cells to differentiated immune cells (47). The ECS exerts an extensive effect on human physiology and is a key regulator of the immune system via CB2Rs. An enhanced role of CB2Rs is well documented in regulating non-neural progenitor cells, hematopoiesis, and bone remodeling. Immune cells such as NK cells, monocytes/ macrophages, and B-lymphocytes express CB2Rs. A clear downregulation of CB2R expression during B-cell differentiation and CB2R upregulation in cell proliferation of activated B-cells further demonstrate the intricate role of CB2R in B-cell development (47).

**Figure 1:**
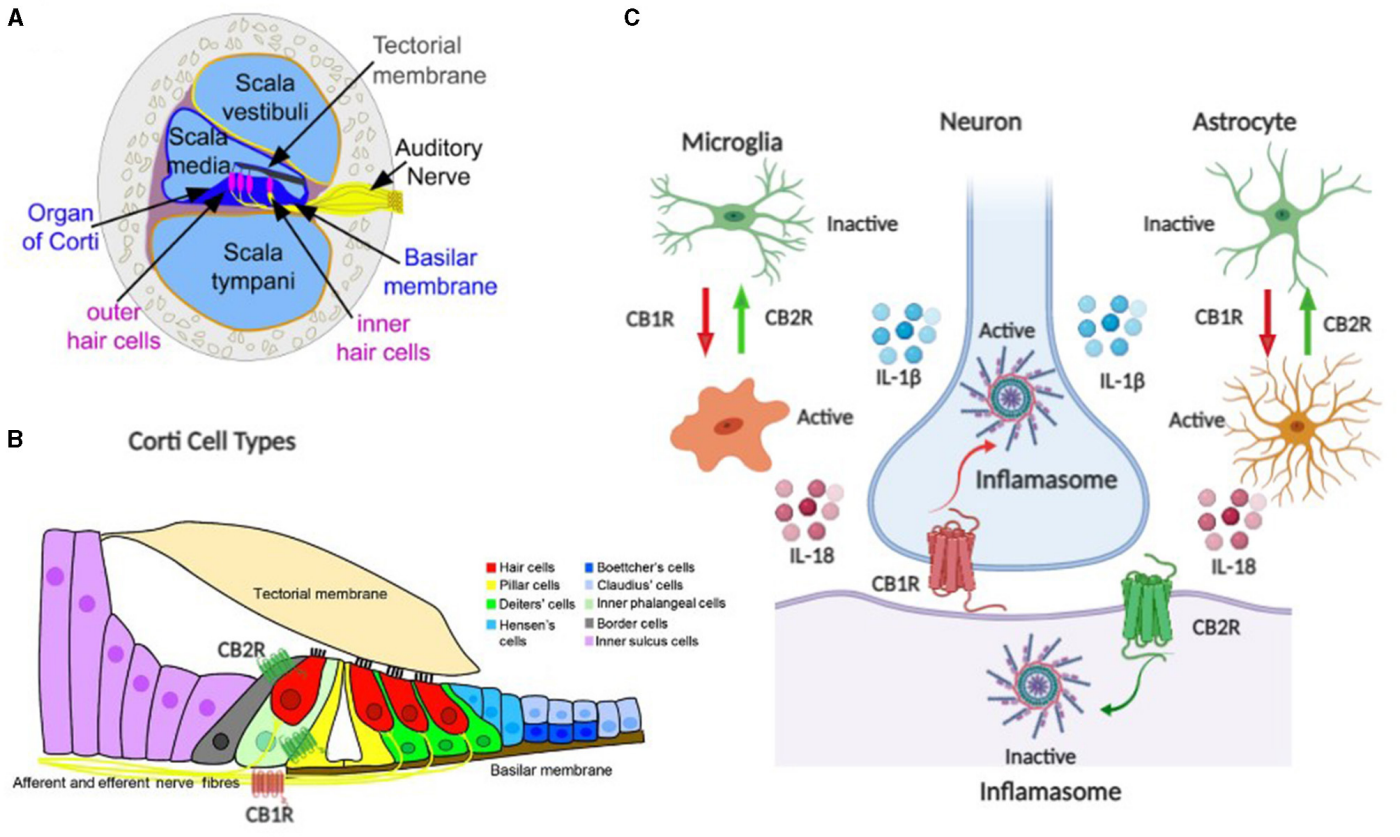
Schematic representation of the endocannabinoid system (ECS) components as neuroimmune targets in auditory system tinnitus. **(A, B)** are components of the auditory ECS–neuroimmune axis including the organ of corti, hair cells, auditory nerve, and corti cell types. **(C)** ECS neuroimmune targets with CB1R and CB2R differential effects in neuronal and glial cells.

Neuroinflammation is an important factor in maintaining homeostasis in the central nervous system against external or internal injury. Furthermore, neuroinflammation is emerging as a key component in the effects of CB2Rs expressed in macrophages, microglia, and neurons (Figure 1), which are key regulators of the immune system (48). Neuroinflammation in sound-processing regions of the brain triggers tinnitus in the mouse model with noise-induced hearing loss, especially in the dorsal cochlear nucleus that has traditionally been associated with the genesis of tinnitus. For example, neurophysiological changes in the auditory cortex were investigated by exposing mice to 8000 Hz tone at 114 dB SPL for 2 h (38, 49). The absence of auditory brainstem response at 50 dB indicated moderate hearing loss. Thus, due to the noise insult neuroinflammation related to the nervous system was evident. They observed that there were elevated levels of pro-inflammatory cytokines/ interleukins, such as IL-1 β, IL-18, TNF-α, and that noise-induced neuroinflammatory response resulted in excitatory-to-inhibitory synaptic balance and caused tinnitus (38). Furthermore, the pharmacological blockade of TNF-α reduced tinnitus. Similar results were reported when rats were injected with salicylate (11). We have identified several B-cell cytokines, some acting as autocrine signals, others as paracrine, or even others as juxtacrine like IL-15, in B-cell lines, suggesting the role of B-cell cytokines in disease progression (50). Although the exact cytokines and mechanisms influencing tinnitus remain unknown, further research using mouse models with cell-specific deletion of CB2R could provide insights into the role of ECS/CB2R in understanding the therapeutic potential of cannabinoid-based treatments by modulating the precise cytokines. We also propose to address the role of CB2R and the associated biochemical changes in the auditory cortex by exposing high-frequency sound to the CB2R cKO, DAT-*Cnr2*, and Cx3cr1-*Cnr2* transgenic mice that were created in Dr. Onaivi's laboratory (46, 48). With brain inflammation associated with tinnitus, ECS/CB2R is a promising potential target to treat neuroinflammation and other diseases including tinnitus. However, a lack of understanding of its complex signaling in cells and tissues complicates the therapeutic exploitation of CB2R as a drug target. We propose studies that will provide insights into the molecular effects of how noise and drug-induced tinnitus could influence the CB2R, thereby revealing the specific cytokine pathways as potential therapeutic targets. Furthermore, pharmacological approaches using CB2R agonist/antagonist as well as CB1R and CB2R mixed agonists like WIN55212–2 will provide a direct role and involvement of the ECS in tinnitus.

## Discussion

### Conclusion and future perspectives

Tinnitus has been well known for millennia, yet pathophysiology is poorly understood, despite progress in technological advances, neuroimaging studies, and various models that have not yielded effective treatment for the subjective type of tinnitus. As suggested by others a paradigm shift may be necessary for tinnitus including neuroimaging approaches to recognizing the effects from specific regions across canonical networks beyond the auditory resting state network (51), and the quantum tunneling ion model. Many facets of tinnitus including the pathophysiology, and objective detection are poorly defined, and currently, there is no sufficient high-level evidence to support effective therapeutic approaches in tinnitus. However, several studies including the quantum tunneling ion model, and lack of objective measures and heterogeneity make tinnitus difficult to treat, but customized acoustic therapy to target known individualized neural pathways is emerging (52). While the current hypothesis on the mechanism of tinnitus is on aberrant activity in the central nervous system with little or no success in therapeutic outcomes, attention to the role of gut microbiota dysbiosis, neuroinflammatory mediators, plasma metabolomics biomarkers along with audiological and vestibular symptoms following SARS-CoV-2 infection may provide further insight in the gut–brain axis crosstalk in understanding the pathophysiology and identifying therapeutic targets in tinnitus (Figure 1). Therefore, future directions may aid in the development of cannabinoid medications for tinnitus with continuous global medical and recreational legalization.

## Data availability statement

The original contributions presented in the study are included in the article/supplementary material, further inquiries can be directed to the corresponding author.

## Author contributions

VB contributed to auditory expertise, and this is one posthumous collaborative perspective of his work. EO contributed to cannabinoid and pharmacological expertise. VS contributed to immunological expertise. All authors contributed to the article and approved the submitted version.

## References

[1] Zheng Y Smith PF. Cannabinoid drugs: will they relieve or exacerbate tinnitus? Cur Open Neurol. (2019) 32:131–6. 10.1097/WCO.0000000000000631 30507635

[2] Young A Ng M. Otoacoustic Emissions. London: StatPearls Publishing (2022). 35593808

[3] Boussaty EC Friedman RA. Million veteran program, clifford re, hearing loss and tinnitus: association for complex hearing disorders in mouse and man. Human Genet. (2022) 141:981–90. 10.1007/s00439-021-02317-9 34318347 PMC8792513

[4] Ibarra D Tavira-Sanchez F Recuero-Lopez M Anthony BW. In-ear medical devices for acoustic therapies in tinnitus treatments, state of the art. Auris Nasus Larynx. (2018) 45:6–12. 10.1016/j.anl.2017.03.020 28438439

[5] Salvari V Korth D Paraskevopoulos E Wollbrink A Ivansic D Guntinas-Licius O. Tinnitus-frequency specific activity and connectivity: A MEG study. NeuroImage: Clin. (2023) 5:103379. 10.1016/j.nicl.2023.103379 36933347 PMC10031544

[6] Hackenberg B Doge J O'Brien K Bohnert A Lackner KJ. Beutel ME. Tinnitus and its relation to depression, anxiety, and stress – A population-based cohort study. J Clin Med. (2023) 12:1169. 10.3390/jcm12031169 36769823 PMC9917824

[7] Gallus S Lugo A Garavello W Bosetti C Santoro E Colombo P. Prevalence and determinants of tinnitus in the Italian adult population. Neuroepidemiology. (2015) 45:12–9. 10.1159/000431376 26182874

[8] Nocini R Lippi G Mattiuzzi C. Impact of COVID-19 pandemic on the worldwide burden of tinnitus. Eur. Arch Oto-Rhino-Laryngology. (2022). 10.21203/rs.3.rs-2060189/v1 36527493 PMC9759037

[9] Jarach CA Lugo A Stival C Bosetti C Amerio A d'Oro LC. The impact of COVID-19 confinement on tinnitus and hearing loss in older adults: Data from the LOST in Lombardia study. Front Neurol. (2022) 13:838291. 10.3389/fneur.2022.838291 35330807 PMC8940241

[10] Scarzynski PH Dziendziel B Gos E Wlodarczyk E Miaskiewicz B Rajchel JJ. Prevalence and severity of tinnitus in otosclerosis: preliminary findings from validated questionnaires. J Int Adv Otol. (2019) 15:277–82. 10.5152/iao.2019.5512 31418718 PMC6750799

[11] Hu SS Mei L Chen JY Huang ZW Wu H. Effects of Salicylate on the inflammatory genes expression and synaptic ultrastructure in the cochlear nucleus of the rats. Inflammation. (2014) 37:365–73. 10.1007/s10753-013-9748-2 24092407

[12] Kameda K Shono T Hashiguchi K Yoshida F Sasaki T Effect Effect of tumor removal on tinnitus in patient with vestibular schwannoma. J Neurosurg. 2010; 112-52. 10.3171/2009.3.JNS081053 19480542

[13] Perin P Tagne AM Enrico P Marino F Cosentino M Pizzala R. Cannabinoids, inner ear, hearing, and tinnitus: A neuroimmunological perspective. Front. Neurol. 11:505995. 10.3389/fneur.2020.505995 33329293 PMC7719758

[14] Clifford RE Maihofer AX Stein MB Ryan AF Nievergelt CM. Novel risk loci in tinnitus and causal inference with neuropsychiatric disorders among adults of European ancestry. JAMA Otolaryngol Head Neck Surg. (2020) 146:1015–25. 10.1001/jamaoto.2020.2920 32970095 PMC7516809

[15] Lopez-Escamez JA Amanat S. Heritability and genetics contribution to tinnitus. Otolaryngol Clin N Am. (2020) 53:501–13. 10.1016/j.otc.2020.03.003 32334875

[16] Qian ZJ Alyono JC. An association between marijuana use and tinnitus. Am J Otolaryngol. (2020) 41:102314. 10.1016/j.amjoto.2019.102314 31732310 PMC7278074

[17] Ghosh S Sheth S Sheehan K Mukherjee D Dhukhwa A Borse V. The endocannabinoid/cannabinoid receptor 2 system protects against cisplatin-induced hearing loss. Front Cell Neurosci. (2018) 12:271. 10.3389/fncel.2018.00271 30186120 PMC6110918

[18] Archie SR Cucullo L. Cerebrovascular and neurological dysfunction under the threat of COVID-19: is there a comorbid role for smoking and vaping? Int J Mol Sci. (2020) 21:3916. 10.3390/ijms21113916 32486196 PMC7312781

[19] Onaivi ES Sharma V. Cannabis for COVID-19: can cannabinoids quell the cytokine storm? Future Sci OA. (2020) 48:FSO0625. 10.2144/fsoa-2020-0124 32974048 PMC7451410

[20] Baig AM. Pathways and pathogenesis of hearing deficits, tinnitus, and vertigo in COVID-19. ACS Chem Neurosci. (2021) 12:4368–70. 10.1021/acschemneuro.1c00706 34780147

[21] Almishaal AA. Comparative study of audiovestibular symptoms between early and late variants of COVID-19. Audio Res. (2022) 12:680–95. 10.3390/audiolres12060065 36546906 PMC9774134

[22] Beukes E Ulep AJ Eubank T Manchaiah V. The impact of COVID-19 and the pandemic on tinnitus: a systematic review. J Clin Med. (2021) 10:2763. 10.3390/jcm10132763 34201831 PMC8268057

[23] Kaliyappan K Chen Y-C Muthaiah VPK. Vestibular cochlear manifestation in COVID-19 cases. Front Neurol. (2022) 13:850337. 10.3389/fneur.2022.850337 35370886 PMC8971520

[24] Degen CV. Mikuteit, Niewolik J, Schroder D, Vahldiek K, Mucke U. Self-reported tinnitus and vertigo or dizziness in a cohort of adult long COVID patients. Front Neurol. (2022) 13:884002. 10.3389/fneur.2022.884002 35547372 PMC9082801

[25] Figueiredo RR Penido NO de Azevedo AA de Oliveira PM de Siqueira AG Figueiredo GMR. Tinnitus emerging in the context of a COVID-19 infection seems not to differ in its characteristics from tinnitus unrelated to COVID-19. Front Neurol. (2022) 13:947179. 10.3389/fneur.2022.974179 36158941 PMC9505692

[26] Daher GS Nassiri AM Vaniichkachkachorn G Carlson ML Neff BA Driscoll CLW. New onset tinnitus in the absence of hearing changes following COVID-19 infection. Am J Otolaryngol Neck Med Surg. (2022) 43:103208. 10.1016/j.amjoto.2021.103208 34536917 PMC8429075

[27] Ahmed SH Waseem S Shaikh TG Qadir NA Siddiqui SA Ullah I. SARS-CoV-2 vaccine-associated tinnitus: a review. Annal Med Surg. (2022) 14:103293. 10.1016/j.amsu.2022.103293 35096388 PMC8788157

[28] Munro KJ Uuus K Almufarrij I Chaudhuri N Yioe V. Persistent self-reported changes in hearing and tinnitus in post-hospitalisation COVID-19 cases. Int J Audiol. (2020) 59:889. 10.1080/14992027.2020.1798519 32735466

[29] Elibol E. Otolaryngological symptoms in COVID-19. Otolaryngological symptoms in COVI-19. Eur Arch Oto-Rhino-Laryngology. (2020) 278:1233–6. 10.1007/s00405-020-06319-7 32875391 PMC7461752

[30] Narozny W Tretiakow D Skorek A. Tinnitus in COVID-19 pandemic. Ear Nose Throat J. (2021) 100:197S−8S. 10.1177/0145561320988364 33470830

[31] Callaway E. Coronavirus variant XBB.1.5 rises in the United States – is it a global threat. Nature. (2023) 613:222–3. 10.1038/d41586-023-00014-3 36624320

[32] Lippi G Sanchis-Gomar F Henry BM. COVID-19 and its long-term sequelae: what do we know in 2023? Pol Arch Int Med. (2023) 14:16402. 10.20452/pamw.16402 36626183

[33] Mizrahi B Sudry T Flak-Manov N Yehezkelli Y Kalkstein N Akiva P. Long-covid outcomes at one year after mild SARS-CoV-2 infection: nationwide cohort study. BMJ. (2023) 380:e072529. 10.1136/bmj-2022-072529 36631153 PMC9832503

[34] Frazier KM Hooper JE Mostafa HH Stewart CM. SARS-CoV-2 virus isolated from the mastoid and middle ear: Implications for COVID-19 precautions. JAMA Otolaryngol Head Neck Surg. (2020) 146:964–6. 10.1001/jamaoto.2020.1922 32701126 PMC7378866

[35] Ali S Kang AK Patel TR Clark JR Perez-Giraldo GS Orban ZS. Evolution of neurologic symptoms in non-hospitalized COVID-19 “long haulers.” Annal. Clin Transl Neurol. (2022) 9:950–61. 10.1002/acn3.51570 35607826 PMC9268866

[36] Xia B Pan X Luo R-H Shen X Li S. Extracellular vesicles mediate antibody-resistant transmission of SARS-CoV-2. Cell Discovery. (2023) 9:2. 10.1038/s41421-022-00510-2 36609376 PMC9821354

[37] Rauscheker JP Leaver AM Muhlau M. Tuning out the noise: Limbic-auditory interactions in tinnitus. Neuron. (2010) 66:819–26. 10.1016/j.neuron.2010.04.032 20620868 PMC2904345

[38] Kotak VC Fujiswa S Lee FA Karthikeyan O Aoki C Sanes DH. Hearing loss raises excitability in the auditory cortex. J Neurosci. (2005) 25:3908–18. 10.1523/JNEUROSCI.5169-04.2005 15829643 PMC1764814

[39] House JW Brackmann DE. Tinnitus: Surgical treatment. Ciba Found Symp. (1981) 85:204–16. 10.1002/9780470720677.ch12 6915835

[40] Zheng Y Back J-H Smith PF Darlington CL. Cannabinoid receptors down-regulation in the ventral cochlear nucleus in an animal model of tinnitus. Hear Res. (2007) 228:105–11. 10.1016/j.heares.2007.01.028 17376618

[41] Premoli M Aria F Bonini SA Maccarinelli G Gianoncelli A Pina SD. Cannabidiol: recent advances and new insights for neuropsychiatric disorders treatment. Life Sci. (2019) 224:120–7. 10.1016/j.lfs.2019.03.053 30910646

[42] Tapasak B Edelmayer L Seidman MD. Endocannabinoid system and otolaryngologist. Otolaryngol Clin N Am. (2022) 55:1101–10. 10.1016/j.otc.2022.06.012 36088164

[43] Narwani V Bourdillon A Nalamada K Manes RP Hildrew DM. Does cannabis alleviate tinnitus? A review of the current literature. Laryngoscope Inv Otolaryngol. (2020) 5:1147–55. 10.1002/lio2.479 33364406 PMC7752070

[44] Roberts LE Eggermont JJ Caspary DM Shore SE. MelcherJR, Kaltenbach JA. Ringing ears: the neuroscience of tinnitus. J Neurosci. (2010) 30:14972–9. 10.1523/JNEUROSCI.4028-10.2010 21068300 PMC3073522

[45] Toal KL Radziwon KE Friedman MA Dent ML. Audiograms, gap detection thresholds, and frequency difference limens in cannabinoid receptor 1 knockout mice Hearing Res. (2016) 332:217–22. 10.1016/j.heares.2015.09.013 26427583 PMC4769947

[46] Liu QR Canseco-Alba A Liang Y Ishiguro H Onaivi ES. Low basal CB2R in dopamine neurons and microglia influences cannabinoid tetrad effects. Int J Mol Sci. (2020) 21:9763. 10.3390/ijms21249763 33371336 PMC7767340

[47] Galve-Roperh I Chiurcchiu V Diaz-Alonso J Bari M Guzman M Maccarrone M. Cannabinoid receptor signaling in progenitor/stem cell proliferation and differentiation. Prog Lipid Res. (2013) 52:633–50. 10.1016/j.plipres.2013.05.004 24076098

[48] Liu QR Canseco-Alba A Zhang HY Tagliaferro P Chung M Dennis E. Cannabinoid type 2 receptors in dopamine neurons inhibits psychomotor behaviors, alters anxiety, depression and alcohol preference. Sci Rep. (2017) 7:17410. 10.1038/s41598-017-17796-y 29234141 PMC5727179

[49] Wang W Zhang LS Zinsmaier AK Patterson G Leptich EJ Shoemaker SL. Neuroinflammation mediates noise-induced synaptic imbalance and tinnitus in rodent models. PLoS Biol. (2019) 17:1–25. 10.1371/journal.pbio.3000307 31211773 PMC6581239

[50] Sharma V. Current perspectives on cytokines for anti-retroviral therapy in AIDS-related to B-cell lymphomas. Curr. Drug Targets Infect, Disord. (2003) 8:137–49. 10.2174/1568005033481178 12769791

[51] Moring JC Husain FT Gray J Franklin C Peterson AL. Resick PA. Invariant structural and functional brain regions associated with tinnitus: a meta analysis. PLoS ONE. (2022) 17:e0276140. 10.1371/journal.pone.0276140 36256642 PMC9578602

[52] Connell JT Bassiouni A Harrison E Laden S O'Brien S Sahpta R. Customized acoustic therapy delivered through a web-based platform – An innovative approach to tinnitus treatment. Clin Otolaryngol. (2023) 48:226–34. 10.1111/coa.14027 36550768

